# The Important Role of Stereotypes in the relation between Mental Health Literacy and Stigmatization of Depression and Psychosis in the Community

**DOI:** 10.1007/s10597-021-00842-5

**Published:** 2021-05-26

**Authors:** Carolin M. Doll, Chantal Michel, Linda T. Betz, Benno G. Schimmelmann, Frauke Schultze-Lutter

**Affiliations:** 1 Department of Psychiatry and Psychotherapy, Faculty of Medicine and University Hospital Cologne, Heinrich-Heine-University, Düsseldorf, Germany; 2 Department of Psychiatry and Psychotherapy, Faculty of Medicine and University Hospital Cologne, University of Cologne, Kerpener Str. 62, 50937 Cologne, Germany; 3 University Hospital of Child and Adolescent Psychiatry and Psychotherapy, University of Bern, Bern, Switzerland; 4 University Hospital of Child and Adolescent Psychiatry, University Hospital Hamburg-Eppendorf, Hamburg, Germany; 5 Department of Psychology and Mental Health, Faculty of Psychology, Airlangga University, Surabaya, Indonesia

**Keywords:** Mental disorders, Mental health literacy, Stereotyping, Stigma, Structural equation model

## Abstract

Increased mental health literacy (MHL) has not reduced stigmatization of people with mental disorder. Thus, we examined the role of stereotypes in the interplay of MHL (correct labelling, causal explanations) and the wish for social distance (WSD) from people with depressive and psychotic symptoms in a community sample of 1526 German-speaking participants in the Swiss ‘Bern Epidemiological At-Risk’ study (age 16–40 years; response rate: 60.1%). Following the presentation of an unlabelled case vignette of depression or psychosis, MHL, stereotypes and WSD were assessed in a questionnaire survey. Their interrelations were studied using structural equation modelling. MHL was not directly linked to WSD, only the psychosocial causal model was directly negatively associated with WSD. Perceived dangerousness particularly increased WSD, this was increased by a biogenetic causal model and decreased by a psychosocial causal model. Awareness-campaigns that, next to biological causes, emphasize psychosocial causes of mental disorders might better reduce stigmatization.

**Supplementary Information:**

The online version contains supplementary material available at 10.1007/s10597-021-00842-5.

## Introduction

Approximately every 4th European adult experiences a mental illness each year (World Health Organization, 2019). In doing so, patients suffer not only from their symptoms and related disabilities, but also experience stigmatization in the community (Alonso et al., 2009; Tsang, 2003; Wahl, 1999) and, similarly, in mental health care facilities (Nyblade et al., 2019; Schulze, 2007). Stigmatization is defined in the World Health Report 2001 as “a mark of shame, disgrace or disapproval which results in an individual being rejected, discriminated against, and excluded from participating in a number of different areas of society” (World Health Organization, 2001, p. 4). Stigma is commonly divided into public stigma, self-stigma and personal stigma. While public stigma is defined as negative stereotypes and prejudice toward people with mental illness held by the community, personal stigma is defined by the individual’s own stereotypes and prejudice (Eisenberg et al., 2009; Griffiths et al., 2004). Based on these definitions, self-stigma occurs when a patient identifies him-/herself with the stigmatized group; thus causing shame, social withdrawal and demoralisation (Corrigan et al., 2009; Corrigan & Shapiro, 2010). Personal stigma, among others, can be measured as the wish for social distance (WSD), i.e., the wish to avoid a specific group, such as people with a mental disorder (Jorm & Oh, 2009).

Many anti-stigma campaigns (Brijnath et al., 2016; Crisp et al., 2004; Henderson et al., 2013; Larkings & Brown, 2018; Reavley et al., 2005) were based on the intuitive assumption that improved Mental Health Literacy (MHL) would reduce discrimination and stigmatization of people with mental disorders (Angermeyer et al., 2009; Hanisch et al., 2016; Hinshaw & Stier, 2008). MHL is defined as the knowledge about symptoms, causes, treatment, and prevention of mental disorders. This includes effective self-help strategies for mild mental problems and first-aid skills to help others (Jorm, 2012). Thus, MHL is frequently considered an important target in campaigns to improve help-seeking for mental problems (Henderson et al., 2013). MHL can vary depending on the mental disorder, for example people were more likely to correctly label symptoms of depression rather than symptoms of schizophrenia (Furnham et al., 2009; Jorm et al., 1997).

However, although MHL and hypothetical help-seeking intentions have steadily increased in the community (Angermeyer & Matschinger, 2005; Angermeyer et al., 2009; Deacon, 2013; Chamberlain et al., 2012; Goldney & Fisher, 2008; Goldney et al., 2005; Jorm et al., 2006; Schomerus et al., 2012), delays in or lack of active help-seeking and stigmatization of people with mental disorder continue to be a serious problem (Angermeyer et al., 2009; Angermeyer et al., 2004; Henderson et al., 2013; Schnyder et al., 2017; Wang et al., 2007).

One reason suggested for this lack of improvement in active help-seeking and attitudes towards people with mental disorders was an unintended consequence of increasing MHL. As part of MHL-improving campaigns, biological factors have frequently been emphasized as a cause of mental illness, in particular depression or schizophrenia (Pescosolido et al., 2010; Schomerus et al., 2012). Some studies also found that people were more likely to attribute schizophrenia to biological causal factors rather than depression (Angermeyer et al., 2015; Dietrich et al., 2004; Von Lersner et al., 2019). While the resulting higher endorsement of a biological model decreased the perception of psychiatric patients as responsible and blameworthy for their problems (Kvaale et al., 2013; Lebowitz & Appelbaum, 2017), it also increased prognostic pessimism, and the perceived unpredictability and dangerousness, i.e., negative stereotypes (Haslam, 2015). Thus, the related decrease in empathy towards psychiatric patients, and the increase in self-blame and personal distress likely intensified the WSD (Haslam, 2015; Kvaale et al., 2013; Larkings & Brown, 2018; Lebowitz, 2019; Rüsch et al., 2010; Von Lersner et al., 2019), thus not reducing but possibly even increasing stigmatization, especially in the case of psychosis (Larkings & Brown, 2018; Read et al., 2006; Schnyder et al., 2018).

**Fig. 1:**
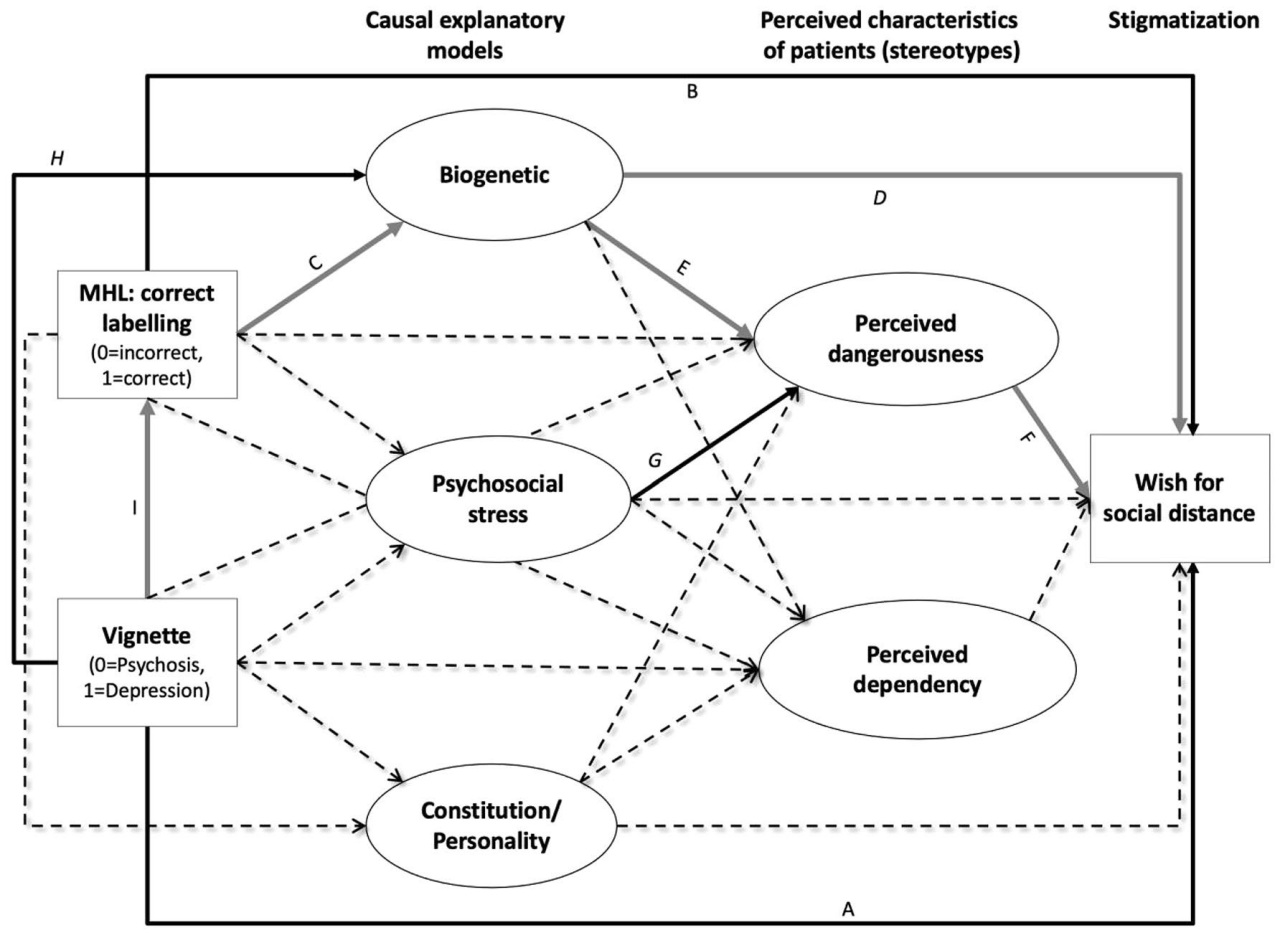
Illustration of associations between causal explanations, stereotypes and WSD reported in the literature. Manifest variables in our model are represented in rectangles, latent ones in ovals. Solid lines indicate reported significant associations (paths) with grey indicates positive and black negative associations; dashed lines indicate paths with no or insignificant reported associations: **A** Independent of any label, the description of a person with symptoms of psychosis was associated with a stronger WSD compared to the description of a person with symptoms of major depression (Angermeyer, Matschinger, et al., 2013; Angermeyer, Millier, et al., 2013; Schomerus et al., 2012; Von Lersner et al., 2019). **B** Good MHL was associated with less pronounced WSD (Angermeyer et al., 2009; Hanisch et al., 2009; Hinshaw & Stier, 2008). **C** Participants with a good MHL more frequently endorse a biogenetic model (Pescosolido et al., 2010; Schomerus et al., 2012). **D** Endorsing a biogenetic model increases WSD (Haslam, 2015; Kvaale et al., 2013; Larkings & Brown, 2018; Lebowitz, 2019; Rüsch et al., 2010; Von Lersner et al., 2019). **E** Endorsing a biological model increases the perceived dangerousness of people with a mental disorder (Haslam, 2015; Kvaale et al., 2013). **F** Perceived dangerousness increases WSD (Angermeyer & Matschinger, 2004; Norman et al., 2008). **G** Endorsing a psychosocial stress model decreases the perceived dangerousness of people with a mental disorder (Schnyder et al., 2018). **H** Schizophrenia is more likely attributed to a biological model than depression (Angermeyer et al., 2015; Dietrich et al., 2004; Von Lersner et al., 2019). **I** Depression is more often correctly labelled than schizophrenia (Furnham et al., 2009; Jorm et al., 1997)

Commonly, these various associations between MHL, personal stigma and stereotypes were studied selectively in separate regression analyses (Angermeyer et al., 2009; Norman et al., 2008; Pescosolido et al., 2010). These studies revealed various, partly contradictory associations between MHL and personal stigma that are summarized in Fig. 1. In particular, the role of biogenetic causal models was ambiguous, as, it was associated with good MHL that is related to lower WSD (Angermeyer, Matschinger, et al., 2013; Angermeyer, Millier, et al., 2013; Schomerus et al., 2012; Von Lersner et al., 2019) and, conversely, was related to both more negative stereotypes (Haslam, 2015; Kvaale et al., 2013) and stronger WSD (Haslam, 2015; Kvaale et al., 2013; Larkings & Brown, 2018; Lebowitz, 2019; Rüsch et al., 2010; Von Lersner et al., 2019).

To resolve such inconsistencies, complex models are needed, such as path analyses or structural equation modelling (SEM) that simultaneously consider complex interrelations of several factors. Yet, these were rarely conducted in this area of research and, if so, with regard to other variables; such as cultural collectivism, professional help-seeking beliefs, help-seeking intentions, healthcare utilisation, emotional reactions to people with mental illness (Altweck et al., 2015; Schnyder et al., 2018; Schomerus et al., 2014; Von Lersner et al., 2019), or in small non-representative or selected samples (Clement et al., 2015; Lanfredi et al., 2019; Trani et al., 2016; Von Lersner et al., 2019).

As SEM models have the advantage to show direct and indirect effects, we examined the interplay between MHL and stereotypes, with respect to personal stigmatization in terms of WSD, in the context of schizophrenia and depression, in a large representative Swiss community sample using a SEM approach (Fig. 1). Better understanding of this complex interplay will help to improve future anti-stigma campaigns, by avoiding potential unintended negative consequences.

## Method

### Study Design and Procedure

Our study was conducted as an add-on study to the ‘Bern Epidemiological At-Risk’ (BEAR) study between June 2011 and June 2015; and participation in the BEAR study was the main eligibility criterion of the add-on study, sufficient language skills in German the only other (Schnyder et al., 2018; Schultze-Lutter et al., 2014; Schultze-Lutter, Michel, et al., 2018; Schultze-Lutter, Schmidt, et al., 2018). Within the BEAR study, 2683 participants, randomly drawn from the population register of the Canton Bern, Switzerland, were recruited for a telephone interview (response rate: 63.4%; Supplementary material eFigure 1) (Schultze-Lutter et al., 2018; Schultze-Lutter, Schmidt, et al., 2018). Because the BEAR study had focussed on the assessment of the prevalence and clinical relevance of clinical high-risk of psychosis criteria and symptoms, inclusion criteria restricted the age range to between 16 and 40 years, i.e., age range with the highest incidence of first-episode psychosis, and excluded people with a past or present psychosis (Schultze-Lutter et al., 2018a, 2018b). Further inclusion criteria were main residency in the Canton Bern, an identified working telephone number, and availability during the recruitment period. Exclusion criteria included insufficient language skills in German, English, French or Spanish (Schultze-Lutter et al., 2018a, 2018b). Compared to the Canton statistics of 16-to-40-year-olds, the BEAR sample was well representative (Schultze-Lutter et al., 2018a, 2018b).

At the conclusion of the telephone interview, 2539 participants in the BEAR study with sufficient knowledge of German (eligibility rate: 94.6%) were asked to participate in the separate add-on study on MHL and attitudes towards people with mental illness. Of these, 2215 participants agreed to additionally participate in the add-on survey (82.4%) and were mailed the questionnaires (details in Schnyder et al., 2018). After a maximum of three reminder calls, 1526 participants returned the questionnaire. Thus, according to the definitions of the American Association for Public Opinion Research (American Association for Public Opinion Research, 2016), both response and cooperation rates of the add-on study were 60.1%, and the overall refusal/non-responder rate was 39.9%. The minor differences between responders and non-responders/refusers to the add-on study were of small effect size at most (Supplementary material eTable1).

Separate verbal informed consent was obtained and recorded from all subjects prior to assessments in both the BEAR study and the add-on study. The authors assert that all procedures contributing to this work comply with the ethical standards of the relevant national and institutional committees on human experimentation and with the Helsinki Declaration of 1975, as revised in 2008, and were approved by the ethical committee of the University of Bern (No. 172/09). Furthermore, all authors certify responsibility.

### Assessments

Demographic and clinical information was assessed as part of the telephone interview (Schultze-Lutter, Michel, et al., 2018; Schultze-Lutter, Schmidt, et al., 2018). For the assessment of MHL (incl. labelling and causal explanations), attitudes towards and perceived characteristics of people with a mental disorder (stereotypes), we used the well-established German questionnaires of Angermeyer and colleagues (Angermeyer & Matschinger, 1996, 1999; Angermeyer et al. 2001; van Brakel, 2006; Link et al., 2004). The questions on causal explanations resulted from a review of the literature at the time and were further refined by first studies using open questions (Angermeyer & Matschinger, 1996; Angermeyer et al. 2001). Stereotypes were assessed according to the scale “stereotype agreement” from the German version of the “Self-Stigma in Mental Illness Scale” (Corrigan et al., 2006; Rüsch et al., 2006) that was slightly modified in the wording of the instruction. The questionnaire starts with an unlabelled case vignette (Angermeyer et al., 2009; Angermeyer et al., 2001, 2004). The two alternatively presented vignettes describe a hypothetical acquaintance with symptoms fulfilling DSM-III-R criteria for either schizophrenia or depression (Supplementary material eText1); their validity was established by blinded rating of five mental health professionals (Angermeyer et al., 2001). The participants were randomly presented either the psychosis vignette or the depression vignette, and, within an open question, first asked to briefly state what they think the person in the vignette is suffering from. These descriptions were subsequently dichotomized as correct when the description included correct name of the disorder or of its constituting symptoms; all other descriptions were labelled as incorrect (Angermeyer & Matschinger, 1999; see Supplementary material eTable 2 for coding examples). Correct labelling was equalled to good overall MHL. Questions regarding 18 possible causal explanations for the behaviour described in the vignette (Table 2) were presented using a five-point Likert scale from 0 = ‘certainly not a cause’ to 4 = ‘certainly a cause’. Furthermore, for the assessment of stereotypes, the participants were presented nine characteristics (Table 3) to be rated according to the degree that they apply to the person described in the vignette on a five-point Likert scale from 0 = ‘definitely not true’ to 4 = ‘definitely true’.

Stigmatization in terms of WSD was assessed according to the Social Distance Scale (Link et al., 1987), self-rating the participant’s willingness to socially interact in seven different situations with the person described in the vignette on a five-point Likert scale from 0 = ‘definitely willing’ to 4 = ‘definitely not willing’ (Angermeyer & Matschinger, 1996). Higher sum scores indicate stronger WSD.

### Statistical Analyses

First, principal component analyses (PCA) with Varimax rotation and pairwise complete observations to deal with missing values were conducted separately on the 18 items on causal explanations, and on the nine items on stereotypes. Resulting factors were examined for their construct validity in terms of the composite reliability using “lavaan” (Rosseel, 2012). Composite reliability is favored over Cronbach’s α when the requirement of t-equivalence (i.e., all items measure the same true value) is violated (Danner, 2015), e.g., in factors composed of items measuring different aspects of a latent construct. However, calculation of the composite reliability requires at least four items per factor (Danner, 2015). Thus, despite the reported biases and limitations of the α coefficient (Cho & Kim, 2015; Revelle & Zinbarg, 2009; Sijtsma, 2009; Sijtsma & van der Ark, 2015), we also calculated Cronbach’s α, which, however, can only be regarded an estimation of the lower boundary of reliability as it tends to underestimate the reliability in factors with few items as well as when t-equivalence is not given (Danner, 2015). For the ordinal nature of items, we used Spearman’s correlation coefficient to construct the correlation matrix. The Kaiser–Meyer Olkin (KMO) measure was used to check the sampling adequacy for the analyses.

Next, we conducted a SEM, which included all associations reported in previous studies (Fig. 1). Missing items (0.03%) were accounted for by using the estimator ‘full information maximum likelihood’ (FIML; Kline, 2011). Based on the results of the PCA and of previous studies (Angermeyer et al., 2004; Schnyder et al., 2018), we defined five latent variables for causal explanations (‘biogenetic’, ‘psychosocial stress’, ‘childhood adversity’, ‘substance abuse’, and ‘constitution/personality’) and two for stereotypes (‘perceived dangerousness’ and ‘perceived dependency’). The variables ‘group’ (depression or schizophrenia vignette), ‘correct labelling’ as a general measure of MHL and ‘WSD’ were modelled as observed binary variables. The pathways from ‘group’ and ‘correct labelling’ via causal explanations and stereotypes to WSD with all possible associations between latent and observed variables were modelled (Fig. 1).

To control for the reported effect of type of mental disorder (Angermeyer et al., 2014; Angermeyer, Matschinger, et al., 2013; Angermeyer, Millier, et al., 2013; McCann et al., 2018; Norman et al., 2012; Sevensson & Hansson, 2016), we included the variable ‘vignette’ in our analysis. Furthermore, for the reported sex differences in stigmatization and MHL (Dey et al., 2020; Hadjimina & Furnham, 2017), we analysed a model with the control variable ‘sex’ (see Supplementary material eFigure 3). However, due to our age restriction to 16- to 40-year-olds, the reported age effect in older people of age 65 + was unlikely to work in our younger sample; thus, we did not include age as a control variable (Mackenzie et al., 2019).

In order to test for mediation effects in the final model, we used “lavaan” (Rosseel, 2012). Thereby, we labelled potential variables in the regression as parameters, so that we could use these parameters to create mediation pathways within the model. The statistical analyses were conducted in SPSS 25.0 and in the R language for statistical computing using the packages “lavaan” (Rosseel, 2012) and “psych” (Revelle, 2018). Throughout, we considered a level of significance of α < 0.05.

## Results

### Sample Characteristics

A similar number of questionnaires with a psychosis (n = 784) and with a depression vignette (n = 742) was returned ($\chi_{(1)}^{2} = 1.1.56$, p = 0.282). Slightly less males than females returned the questionnaire (Table 1). The average age of participants was 31 years; most of them were Swiss, unmarried, and normally employed, and had a short cycle tertiary education or Master degree (Table 1). Every 8th participant had met criteria for a current non-psychotic axis I disorder in the telephone interview (Table 1), this number going down to every 14th participant (n = 108; 7.1%) when excluding specific phobias. Almost half of the sample reported a 1st- or 2nd-degree family member with suspected or diagnosed mental disorder—mostly with an affective, rarely a psychotic disorder (Table 1). Clinical and sociodemographic variables did not differ significantly between responders of the two vignettes (Table 1).

**Table 1:** Sample characteristics of the responders to the add-on study (N = 1526) according to the case vignette of the questionnaire

	Depression (n = 742)	Psychosis (n = 784)	Total sample (N = 1526)	Statistics U/χ^2^(df); Pearson’s r/Cramer’s V
Sex, n (%) male	353 (47.8)	365 (46.7)	718 (47.2)	$\chi_{(1)}^{2}$ = 0.162, p = 0.687, V = 0.010
Age: median (mean ± SD)	33.84 (31.10 ± 7.3)	33.91 (31.53 ± 7.22)	33.86 (31.32 ± 7.27)	U = 277 609, p = 0.200, r = − 0.030
Nationality, n (%) Swiss	706 (95.5)	749 (95.9)	1455 (95.7)	$\chi_{(1)}^{2}$ = 0.126, p = 0.723, V = 0.009
Highest educational level (ISCED 2011)^a^, n (%)				$\chi_{(6)}^{2}$ = 7.452, p = 0.281, V = 0.070
Primary education (1)	0	0	0	
Lower secondary education (2)	24 (3.2)	18 (2.3)	42 (2.8)	
Higher secondary education (3)	13 (1.8)	13 (1.7)	26 (1.7)	
Post-secondary non-tertiary education (4)	4 (0.5)	9 (1.2)	13 (0.9)	
Short cycle tertiary education (5)	405 (54.8)	390 (49.9)	795 (52.1)	
Master’s or equivalent level (7)	247 (33.4)	301 (38.5)	548 (35.9)	
Doctoral or equivalent level (8)	12 (1.6)	12 (1.5)	24 (1.6)	
Employment, n (%)				$\chi_{(3)}^{2}$ = 2.698, p = 0.441, V = 0.042
Unemployed	16 (2.2)	9 (1.2)	25 (1.6)	
Protected employment	1 (0.1)	2 (0.3)	3 (0.2)	
Temporarily/self-employed	9 (1.2)	9 (1.2)	18 (1.2)	
Normal employment, in school/training	713 (96.5)	761 (97.4)	1474 (97.0)	
Marital status, n (%)				$\chi_{(2)}^{2}$ = 0.259, p = 0.998, V = 0.013
Unmarried	393 (53.2)	406 (52.0)	799 (52.6)	
Married or registered partnership	320 (43.3)	348 (44.6)	668 (43.9)	
Separated/Divorced/Widowed	25 (3.4)	26 (3.3)	51 (3.3)	
Current non-psychotic axis-I disorder^b^, n (%)	97 (13.1)	95 (12.2)	192 (12.6)	$\chi_{(1)}^{2}$ = 0.318, p = 0.573, V = 0.014
Family member with a mental disorder, n (%)				
Affective disorder	185 (25.1)	216 (27.7)	401 (26.4)	$\chi_{(1)}^{2}$ = 1.238, p = 0.266, V = 0.029
Psychotic disorder	23 (3.1)	25 (3.2)	48 (3.2)	$\chi_{(1)}^{2}$ = 0.007, p = 0.993. V = 0.002

^a^According to International Standard Classification of Education (ISCED) (UNESCO Institute for Statistics, 2012)

^b^Acording to Mini-International Neuropsychiatric Interview

### Factors of Causal Explanations and Stereotypes

The KMO measure indicated excellent or “meritorious” (Kaiser, 1974) sampling adequacy for the analyses (KMO = 0.79 and KMO = 0.78), and all KMO values for individual items were > 0.65 in the first and > 0.57 in the second PCA, and therewith above the threshold for acceptability of 0.5 (Schneeweiss & Mathes, 1995). Bartlett’s test of sphericity ($\chi_{(153)}^{2} = 6073.26$, p < 0.001 and $\chi_{(36)}^{2} = 3723.98$, p < 0.001) indicated that correlations between items were sufficiently large for PCA (Schneeweiss & Mathes, 1995). In the PCA of the 18 causal explanations items, five independent factors (‘psychosocial stress’, ‘childhood adversity’, ‘biogenetic’, ‘substance abuse’ and ‘constitution/personality’) had an eigenvalue over Kaiser’s criterion of 1 and explained 55% of the variance (Table 2). In the second PCA, two independent factors (‘perceived dangerousness’, ‘perceived dangerousness’) had an eigenvalue over Kaiser’s criterion of 1 and explained 53% of the variance (Table 3). With regard to the internal consistency of the factors, the two largest factors, ‘psychosocial stress’ and ‘perceived dangerousness’, had satisfactory to good composite reliability values above 0.70 (Hair et al., 2019). The two other factors of four items each, ‘constitution/personality’ and ‘childhood adversity’, were well or almost acceptable (Hair et al., 2019; see Table 2). Cronbach’s α, for the factors with less than four items, indicated lower estimations within the range of “poor” and “questionable” reliability for ‘substance abuse’ and ‘biogenetic’, and just within the “unacceptable” range for ‘perceived dependency’ (see Tables 2 and 3).

**Table 2:** Results of the principal component analysis (PCA) of 18 questions regarding 18 possible causal explanations for the behaviour described in the vignette (n = 1526), and the internal consistency of the factors (composite reliability and Cronbach’s α)

Items	Factor 1: Psychosocial stress	Factor 2: Substance abuse	Factor 3: Constitution/personality	Factor 4: Childhood adversity	Factor 5: Biogenetics
Work-related stress	0.80				
Too high self-expectation	0.71				
Problems or sorrows in family	0.68				
Daily hustles	0.67				
Severe or very stressful life event	0.57				
An unconscious conflict	0.50				
Medication or drug abuse		0.81			
Alcohol abuse		0.79			
Weak will			0.74		
Weak constitution			0.68		
Immoral lifestyle			0.59		
God’s will			0.42		
Grown up in a broken home				0.80	
Lack of parental affection				0.76	
Little support others				0.43	
Spoiling or over-protective parents				0.57	
Heredity					0.75
Brain disease					0.55
Eigenvalue	2.88	1.89	1.87	1.81	1.50
Composite reliability^a^	0.77	–	0.59	0.67	–
Cronbach’s α	0.70	0.60	0.60	0.64	0.55

Only factor loading > 0.40 are displayed in descending order per factor (causal explanations). The instruction this item is as follows “Now, we would like to know your opinion about the cause of problems like the one described above. For your answers, a 5-point response scale is provided. Please, tick for every possible cause to what extent this might be the cause of such a problem.” Rating for each characteristic is done on a 5-point Likert scale ranging from “is certainly one of the causes” to “is certainly not a cause”

^a^In empirical research, values between 0.60 and 0.70 are considered “acceptable,” values between 0.70 and 0.90 range from “satisfactory” to “good” (Hair et al., 2015)

**Table 3:** Results of the principal component analysis (PCA) of 9 questions regarding the characteristics of the person described in the vignette (n = 1526), and the internal consistency of the factors (composite reliability and Cronbach’s α)

Items	Factor 1: Dangerous/unpredictable	Factor 2: Dependent/needy
Dangerous	0.78	
Lacking self-control	0.76	
Frightening	0.75	
Unpredictable	0.74	
Aggressive	0.70	
Strange	0.67	
Dependent on others		0.82
Helpless		0.73
Needy		0.49
Eigenvalue	3.27	1.50
Composite reliability^a^	0.83	–
Cronbach’s α	0.83	0.47

Only factor loading > 0.40 are displayed in descending order per factor (stereotype). The instruction this item is as follows “Now we would like to get to know what characteristics you think apply to this person. Please, tick with each characteristic of the list to what extent it applies or not.” Rating for each characteristic is done on a 5-point Likert scale ranging from “certainly applies” to “certainly not applies”

^a^In empirical research, values between 0.60 and 0.70 are considered “acceptable,” values between 0.70 and 0.90 range from “satisfactory” to “good” (Hair et al., 2015)

### Association Between Correct Labelling, Type of Vignette, Causal Explanations and Personal Attributions on Stigmatizing

**Fig. 2:**
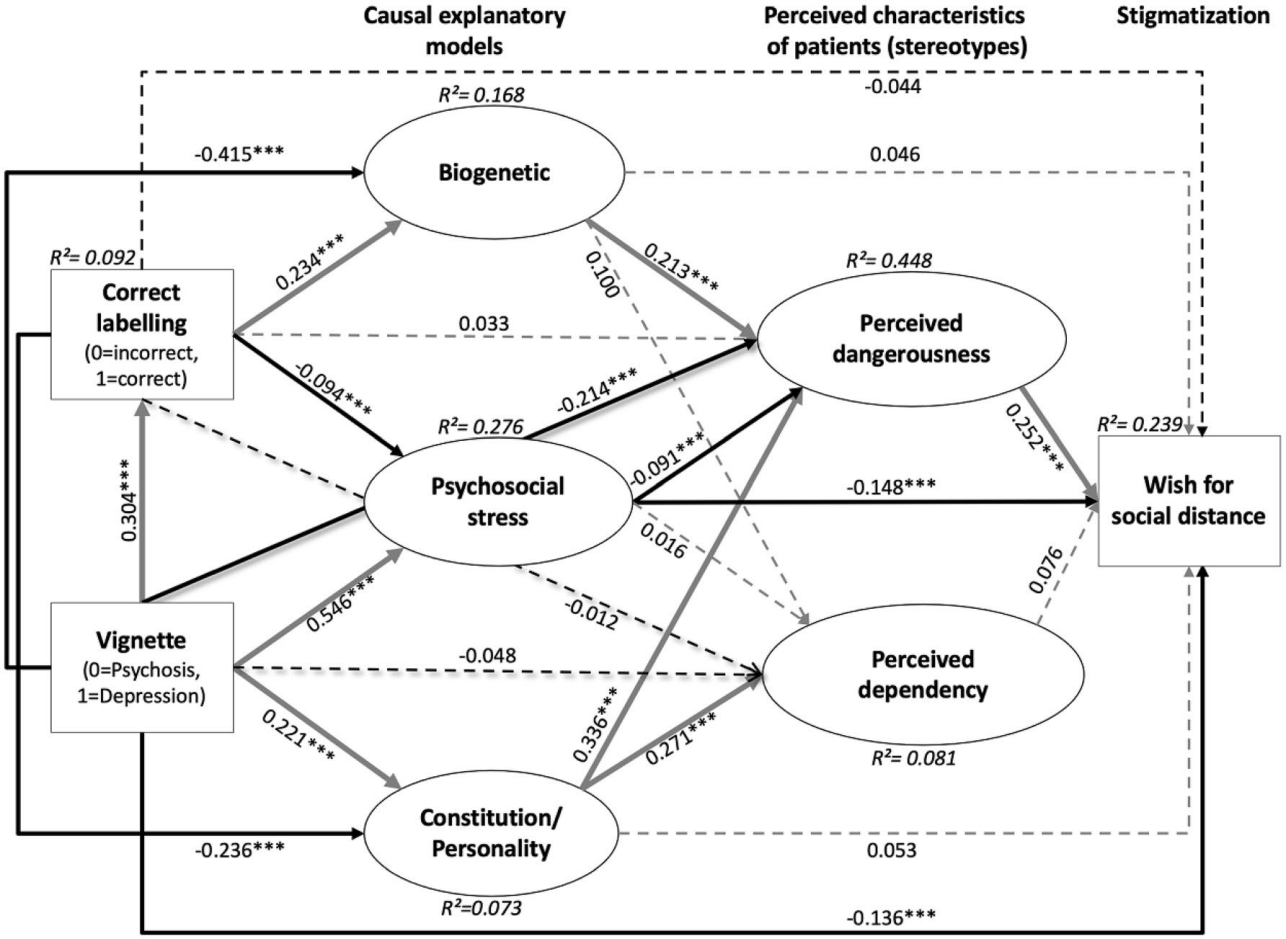
Final model of associations between causal explanations, stereotypes and WSD (n = 1526) with standardized path coefficients. Model fit indices: $\chi_{23(1)}^{2}$ = 1427.895 with p < 0.001, CFI = 0.864; SRMR = 0.052; RMSEA = 0.058 (90%CIs = 0.055, 0.061); PNFI = 0.705. ***p ≤ 0.001; explained variance (R^2^) for each endogenous variable in italics. Manifest variables are represented in rectangles, latent ones in ovals. Solid lines indicate significant paths, dashed lines indicate non-significant paths; in doing so, grey indicates positive, black negative correlations

In the initial SEM that included all 5 causal models, the factors ‘childhood adversity’ (R^2^ = 0.01) and ‘substance abuse’ (R^2^ = 0.02) missed crucial thresholds for good model fit because of their low R-square (see Supplementary material eFigure 2). Thus, they were removed, and only three causal models (‘psychosocial stress’, ‘biogenetic’, ‘constitution/personality’) were taken into the final SEM (Fig. 2, Supplementary material eTable 3).

For the final SEM, the fit indices RMSEA and its 90% confidence intervals, and SRMR were in line with recommended values (≤ 0.06, not containing 0.08, and ≤ 0.08, resp.) suggesting good model fit to data (Hooper et al., 2011) (Fig. 2). The PNFI value was 0.705. Yet, the χ^2^-statistic became significant. The CFI was below the recommended value of ≥ 0.95 (Hooper et al. 2008; Kline, 2011), suggesting possibly insufficient fit. However, two severe problems limit the use of the χ^2^-statistic: (1) severe violations of the assumption of multivariate normality may result in model rejections even of properly specified models; and (2) being essentially a statistical significance test, it is sensitive to sample size and nearly always rejects the model in large samples like ours (Hooper et al., 2008). A problem of the CFI is its assumption of all latent variables being uncorrelated/independent; thus, it is less reliable in models like ours that violate this assumption, as demonstrated by the significant intercorrelations of factors (Supplementary material eTable 4) (Hooper et al., 2008).

Regarding the other fit indices, RMSEA, which measures how well the model, including unknown but optimally chosen parameter estimates, would fit the sample’s covariance matrix, is increasingly “regarded as one of the most informative fit indices” (Hooper et al., 2008, p. 54). Despite its sensitivity to the number of estimated parameters in the model and, relatedly, its favouring of parsimonious models, this important fit index indicated good fit of our model even in the face of its complex, non-parsimonious nature. Furthermore, the SRMR also indicated good fit. Because Hu and Bentler’s ‘2-index presentation strategy’ suggests that a model should be regarded as well fitting, if both RMSEA and SRMR indicate acceptable fit (Hooper et al., 2008), we considered the fit of our model (Fig. 2) acceptable overall.

In line with earlier reports (Fig. 1), the type of vignette was associated with WSD, whereby the psychosis vignette was related to stronger WSD (Fig. 2). Additionally, the type of vignette was significantly related to correct labelling, all causal models and ‘perceived dangerousness’ but not ‘perceived dependency’. In doing so, the psychosis vignette increased the likelihood to endorse a biogenetic model and to perceive the illustrated person as dangerous, while the depression vignette was more likely correctly labelled, and explained by psychosocial stress or constitution/personality-related causes. Unexpectedly, the path from correct labelling as a general measure of good MHL to WSD was not significant, and neither were those between correct labelling and stereotypes. In order to examine an indirect effect of correct labelling on stereotypes via causal models, we tested the pathway “correct labelling—biogenetic—perceived dangerousness” that, however also remained insignificant (p = 0.219). Thus, correct labelling had neither direct nor indirect effects on WSD in our model. Yet, again in line with earlier reports, correct labelling increased endorsement of a biogenetic and non-endorsement of a psychosocial stress or constitution/personality-related causal model. As expected, endorsement of a biogenetic model was related to more perceived dangerousness that intensified WSD. However, contrary to the literature, the biogenetic model was not directly related to WSD. Furthermore, it was also not indirectly related to WSD via perceived dangerousness, as this indirect effect also remained insignificant (p = 0.154). Contrary to the biogenetic model, the constitution/personality-related causal model was negatively associated with correct labelling, and its endorsement not only increased perceived dangerousness but also perceived dependency. Like the biogenetic model, the constitution/personality-related model was also not indirectly related to WSD via perceived dangerousness, as this indirect effect on WSD was also not significant (p = 0.756). Of all causal models, the psychosocial stress model was the only one directly and negatively related to WSD. This positive effect of psychosocial stress-related causal models also worked via reducing the likelihood of perceiving patients as dangerous (p < 0.001) (Fig. 2).

With regard to the impact of sex (see Supplementary material eFigure 3), compared to men, women labelled the vignette correctly more often, were more likely to relate the described disorder to a biogenetic or psychosocial stress model, and were less likely to assume constitution/personality as cause for the mental disorder, and to regard the person in the vignette as ‘perceived dependency’. Despite these significant sex differences in MHL in particular, no significant sex differences revealed for perceived dangerousness or WSD; and the associations of the overall model (Fig. 2) were replicated.

## Discussion

To the best of our knowledge, this study is the first to examine the associations of MHL, stereotypes and stigma in one complex model. While most of its paths had earlier been described, our model sheds light on some of the apparently conflicting results in the literature, in particular the association of good MHL with low WSD as a measure of stigmatization on the one hand (Angermeyer et al., 2009; Hanisch et al., 2016; Hinshaw & Stier, 2008) and, on the other hand, the association of good MHL with endorsement of a biogenetic model (Pescocolido et al., 2010; Schomerus et al., 2012) that, in turn, is associated with a stronger WSD (Haslam, 2015; Kvaale et al., 2013; Larkings & Brown, 2018; Lebowitz, 2019; Rüsch et al., 2010; Von Lersner et al., 2019). Our model now indicates that this apparent contradiction may have resulted from (incorrectly) assuming a direct link between MHL and WSD when important mediators such as stereotypes had not been considered.

In our model, MHL had little effect on WSD as especially evidenced by the non-significant direct paths to WSD from correct labelling and endorsement of a biogenetic causal model. Rather, the symptomatology of the mental illness (i.e., the case vignettes) and the perceived characteristics of the affected person (i.e., the stereotypes) were determinants of WSD.

Anti-stigma and awareness campaigns to improve help-seeking for mental problems and mental health first aid trainings have commonly focused on improving MHL (Angermeyer et al., 2009; Brijnath et al., 2016; Corrigan, 2016; Crisp et al., 2004; Hanisch et al., 2016; Henderson et al., 2013; Hinshaw & Stier, 2008; Larkings & Brown, 2018; Morgan et al., 2018; Sartorius & Schulze, 2005). Yet, although MHL has steadily increased in the community, in particular in terms of increased endorsement of biogenetic causal models, delays in or lack of help-seeking, and stigmatization of people with mental disorder, have improved less- or sometimes even worsened, thus, remaining a serious problem (Angermeyer et al., 2009; Angermeyer, Matschinger, et al., 2013; Angermeyer, Millier, et al., 2013; Corrigan, 2016; Deacon, 2013; Henderson et al., 2013; Larkings & Brown, 2018; Pescosolido et al., 2010; Schomerus et al., 2012). Accordingly, an earlier SEM analysis of our data already demonstrated negative effects of biogenetic and also constitution/personality-related causal models on active help-seeking via a dangerous stereotype and WSD (Schnyder et al., 2018). Yet, this SEM had not studied any direct effect on WSD other than that of the dangerous stereotype (Schnyder et al., 2018). Our study now further supports the unintended effect of improved MHL in terms of correct labelling of the vignette and, relatedly, endorsement of a biogenetic causal model that increased the perception of patients with mental disorder as dangerous and unpredictable. This is in accordance with previous reports of a positive association between endorsement of a biogenetic causal explanation, and the perception of people with a mental disorder as more dangerous (Kvaale et al., 2013; Larkings & Brown, 2018; Pescosolido et al., 2010; Read, 2007; Read & Harré, 2001; Schnyder et al., 2018).

To control for differences related to symptomatology, the vignette was included as a control variable in the model. As in other studies, in the schizophrenia vignette group a biogenetic causal explanation was more likely compared to the depression vignette group (Angermeyer et al., 2015; Dietrich et al., 2004; Von Lersner et al., 2019). The depression vignette group, in turn, more often endorsed a psychosocial explanation. Overall, participants expressed a higher WSD to the person depicted in the psychosis vignette compared to the person in the depression vignette. Thus, in light of the missing direct effect of correct labelling on WSD and with regard to the classic debate of whether behaviours/symptoms or label formed the basis of stigma, our results indicate a major role of symptoms not label (Pescosolido, 2013).

Interestingly, correct labelling was significantly related only to causal explanations but not to stereotypes, having likely a weaker effect on both stereotypes and WSD, compared to symptoms. Hence, our results support earlier findings of psychiatric terminology, i.e., correct labels, not having a direct impact on attitudes toward mental illness (Mann & Himelein, 2004). They also support earlier reports that (illustrated) symptoms play a significant role, with psychotic symptoms being more stigmatized than depressive symptoms (Mann & Himelein, 2004; Norman et al., 2008). Earlier, it was suggested that stigmatization is the worst when the disorder is severe, unfamiliar and, most importantly, socially debilitating, because lay people would focus on visible aspects of social disability (Gaebel et al., 2006). And indeed, the illustrated psychosocial disability was worst in the psychosis vignette compared to the depression vignette (see Supplementary material eText1). This indicates a necessity to prevent development of severe symptoms and psychosocial functional impairment in order to avoid stigmatization, thus reinforcing the view of the WHO that effective prevention of mental disorders can “change the way mental disorders are looked upon by society” (World Health Organization, 2004, p. 3).

Aside from a less severe symptomatology, the only factor with a potential to reduce WSD was endorsement of a psychosocial causal model. This became more apparent in the depression vignette and incorrect labelling of the vignette, mostly of the psychosis vignette (Angermeyer et al., 2009). Furthermore, endorsing a psychosocial causal model reduced perceived dangerousness that increased WSD. Taken together, our findings support critique on awareness campaigns that primarily convey a medical, biological etiological model of mental disorders (Lebowitz, 2019; Longdon & Read, 2017; Schomerus et al., 2014). This critique was based on the “substantial evidence that campaigns based on the "medical model" (such as the "mental illness is an illness like any other" approach) are not only ineffective, but can actually compound the problem” (Longdon & Read, 2017, p. 24). Supporting earlier recommendations, our results support calls for a stronger role of psychosocial explanatory models in MHL-supporting campaigns, in order to promote more positive and tolerant attitudes towards, and inclusion of psychiatric patients (Longdon & Read, 2017; Pescosolido et al., 2010). However, endorsing a biogenetic model was reported to increase help-seeking intentions and support of psychopharmacological and psychotherapeutic treatments (Arboleda-Flórez & Stuart, 2012; Lebowitz & Appelbaum, 2017; Pescosolido et al., 2010), possibly via an underlying fear, reflecting a desire for protection against people with mental illness (Schnyder et al., 2018; Speerforck et al., 2017). Thus, awareness campaigns with a stronger focus on psychosocial causes might bring about the unintended consequence of supporting non-professional help-seeking recommendations (Altweck et al., 2015). In order to escape this vicious circle of unintended consequences, future studies of awareness campaigns should further examine the reported differential effects of biological “brain disease” and genetic “heredity” causal models in relation to different disorders, and their defining and accompanying symptoms, in order to find the most advantageous balance between psychosocial and biogenetic causal models, to optimize beneficial effects on both stigmatization and help-seeking (Speerforck et al., 2014). Such research that cross-sectionally and, importantly, longitudinally examines the interplay of different aspects of MHL and attitudes towards people with mental disorders on both stigmatization and help-seeking using appropriate measures (such as SEM or network analyses) is clearly needed, and might solve the dilemma between the potential stigma-increasing effect of biogenetic models, and the potential help-seeking-reducing effect of psychosocial models.

### Strengths and Limitations

Our study has several strengths and limitations. Among the strengths are clearly the large sample size and the use of SEM as a means to simultaneously consider a multitude of direct and indirect associations.

Among the limitations is a CFI below the recommended value of ≥ 0.95 (Hooper et al. 2008; Kline, 2011), which indicates a possibly insufficient fit. However, the CFI is sensitive to sample size and nearly always rejects the model in large samples like ours (Hooper et al., 2008). Other fit indices (RMSEA and SRMR), however, indicated a good model fit according to Hu and Bentler’s ‘2-index presentation strategy’ (Hooper et al., 2008) This strategy suggests that a model should be regarded as well fitting, if RMSEA and SRMR indicate acceptable fit—like they do in our model. Another limitation of the study might be the partly low internal consistency of the factors that, however, seems mostly related to methodological factors, such as inclusion of few items and/or the fact that included items frequently reflect different aspects of an underlying construct, i.e., are not t-equivalent (Danner, 2015). Thus, while the composite reliability had certainly been the method of choice, in factors with less than three items, only Cronbach’s α could be calculated as an estimate of the lower boundary of reliability. Thus, the true reliability of the factors that all seem clinically plausible is likely higher and in no case unacceptable. Another limitation is the restriction of the sample to German-speaking people of mainly Middle-European background aged 16 to 40. As cultural and also age-related differences with respect to MHL and stigmatization were reported, conclusions drawn from our results may primarily be relevant to European health care systems and to young adults (Altweck et al., 2015; Angermeyer et al., 2016; Nersessova et al., 2019; Pescosolido, 2013; Von Lersner et al., 2019). Yet, the replication of several well-known findings in a complex modelling set-up might also provide evidence for the generalizability of these associations across the Western culture. And the examined age-group represents that of the highest incidence of mental disorders (Pedersen et al., 2014), and, consequently, the age group in which WSD might have its most adverse effects on first help-seeking for mental disorder.

Furthermore, a number of potential moderators on WSD were not considered in our model, e.g., (level of) familiarity with mental illness (Angermeyer et al., 2004; Kasow & Weisskirch, 2010), personal values (Norman et al., 2008), and perceived social norms (Norman et al., 2008). In regards to familiarity—either by own illness or mental illness of a family member, friend or colleague, we had only assessed family history of first- and second-degree biological relatives and only current mental disorders of the participant. Thus, we would have missed familiarity by mental disorders of other well-known people and by own past mental disorder. To avoid introducing a systematic assessment bias, we therefore refrained from including a latent variable ‘familiarity’. A limitation that our study shares with most other interview- or questionnaire-based studies on this topic is the possibility of systematic response biases such as social desirability. Yet, the results are well in line with earlier reported single association between the examined variables, and thus, point towards only minor response biases. Further, in line with the criteria employed by Sastre-Rus et al. (2019), the results indicate that the questionnaires can be assumed to have a strong level of evidence for good quality assessment, supporting earlier notions of them as good-quality instruments to assess attitudes and beliefs about mental illness (Link et al., 2004; van Brakel, 2006).

Overall, our results challenge the view that an improved MHL will unequivocally reduce stigmatization and discrimination of people with mental disorder, especially when biological models are emphasized. Rather, preventive approaches that reduce symptom exacerbation, in combination with education about psychosocial causes, as well as the causes and interplay of symptoms in public campaigns (Schultze-Lutter, Michel, et al., 2018; Schultze-Lutter, Schmidt, et al., 2018), might reduce stigmatizing stereotypes best while still facilitating help-seeking.

## Supplementary Information

Below is the link to the electronic supplementary material.

Supplementary file1 (DOC 27 kb)

Supplementary file2 (DOC 9934 kb)

Supplementary file3 (DOC 13129 kb)

Supplementary file4 (DOC 13301 kb)

Supplementary file5 (DOC 49 kb)

Supplementary file6 (DOC 34 kb)

Supplementary file7 (DOC 73 kb)

## Data Availability

The anonymised data-set is available from the corresponding author on reasonable request.
