# Supplementary material for: The Important Role of Stereotypes in the relation between Mental Health Literacy and Stigmatization of Depression and Psychosis in the Community
Source: Community Ment Health J. 2021 May 26;58(3):474–86. doi: 10.1007/s10597-021-00842-5 (PMC8860791; doi:10.1007/s10597-021-00842-5)
Supplement: Supplementary file 1 — Supplementary file1 (DOC 27 kb) [file 10597_2021_842_MOESM1_ESM.doc]

**eText 1.** English translation (Norman, Sorrentino, Windell, & Manchanda, 2008) of the original German vignettes, which were used in our study.

**Depression vignette, unlabelled:**

Imagine that you know the following about an acquaintance (AB) with whom you occasionally spend your leisure time. Within the past 2 months, AB has changed in nature. In contrast to previously, AB is down and sad without being able to give a concrete reason for feeling low. AB appears serious and worried. There is no longer anything that will make AB laugh. AB hardly ever talks, and if AB says something, AB speaks in a low tone of voice about the worries AB has with regard to AB’s future. AB feels useless and has the impression AB does everything wrong. All attempts to cheer AB up have failed. AB lost all interest in things and is not motivated to do anything. AB complains of often waking up in the middle of the night and not being able to get back to sleep. By the morning, AB feels exhausted and without energy. AB says that AB encounters difficulty in concentrating on AB’s job. Unlike before, everything takes AB a very long time to do. AB hardly manages AB’s workload. As a consequence, AB has already been summoned to AB’s boss. AB has now sought professional help and was told AB appears to be suffering from depression.

**Psychosis vignette, unlabelled:**

Imagine that you know the following about an acquaintance (AB) with whom you occasionally spend your leisure time. In the past months, AB appears to have changed. More and more, AB has retreated from their friends and colleagues, up to the point of avoiding them. If someone managed to involve AB in a conversation, AB would only talk about whether some people have the natural gift of reading other people’s thoughts. This question became AB’s sole concern. In contrast with AB’s previous habits, AB has stopped taking care of their appearance and looked increasingly untidy. At work, AB seemed absent-minded and frequently made mistakes. As a consequence, AB has already been summoned to their boss.

Finally, AB stayed away from work for an entire week without an excuse. Upon their return, AB seemed anxious and harassed. AB now reports being absolutely certain that people cannot only read other people’s thoughts but also directly influence them. AB was, however, unsure who would steer ABs thoughts. AB also said that, when thinking, AB was continually interrupted. Frequently, AB would even hear those people talk to AB, and they would give AB instructions. Sometimes, they would also talk to each other and make fun of whatever AB was doing at the time. AB said that the situation was particularly bad at AB’s apartment. At home, AB would really feel threatened, and would be terribly scared. Hence, AB had not spent the night at AB’s place for the past week, but rather had hidden in hotel rooms and hardly dared to go out. AB has now sought professional help and was told AB appears to be suffering from schizophrenia

**Reference**

Norman, R. M., Sorrentino, R., Windell, D., & Manchanda, R. (2008). Are personal values of importance in the stigmatization of people with mental illness? *Can J Psychiatry, 53*(12), 848-856.
