# Supplementary material for: The Important Role of Stereotypes in the relation between Mental Health Literacy and Stigmatization of Depression and Psychosis in the Community
Source: Community Ment Health J. 2021 May 26;58(3):474–86. doi: 10.1007/s10597-021-00842-5 (PMC8860791; doi:10.1007/s10597-021-00842-5)
Supplement: Supplementary file 2 — Supplementary file2 (DOC 9934 kb) [file 10597_2021_842_MOESM2_ESM.doc]

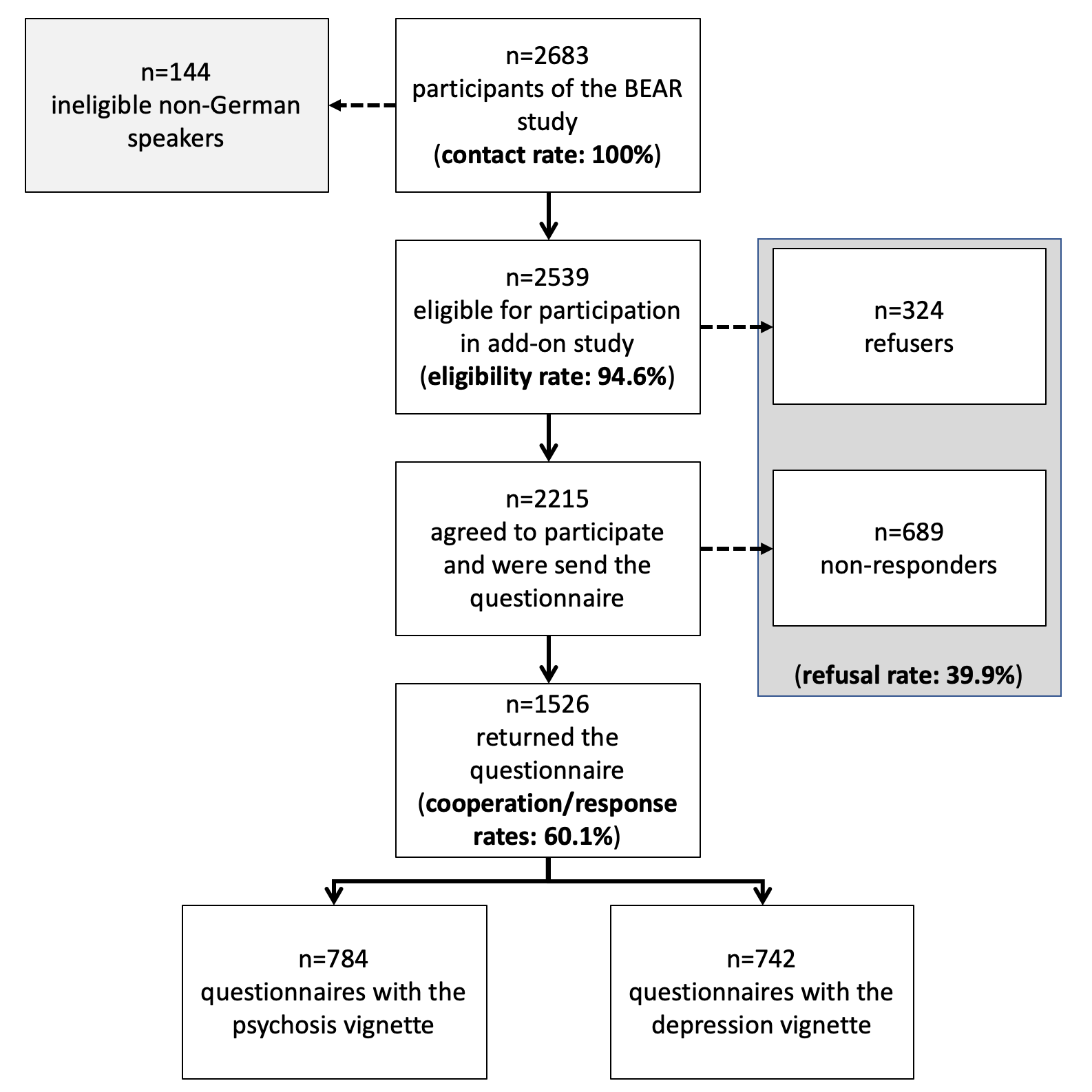


eFigure 1. Recruitment process of the add-on study to the BEAR study according to the AAPOR Outcome Rate Calculator, version 3.1.(AAPOR American Association for Public Opinion Research, 2016)

**Reference**

American Association for Public Opinion Research. (2016). Standard Definitions: Final Dispositions of Case Codes and Outcome Rates for Surveys. 9 edition. AAPOR.
