# Supplementary material for: The Important Role of Stereotypes in the relation between Mental Health Literacy and Stigmatization of Depression and Psychosis in the Community
Source: Community Ment Health J. 2021 May 26;58(3):474–86. doi: 10.1007/s10597-021-00842-5 (PMC8860791; doi:10.1007/s10597-021-00842-5)
Supplement: Supplementary file 4 — Supplementary file4 (DOC 13301 kb) [file 10597_2021_842_MOESM4_ESM.doc]

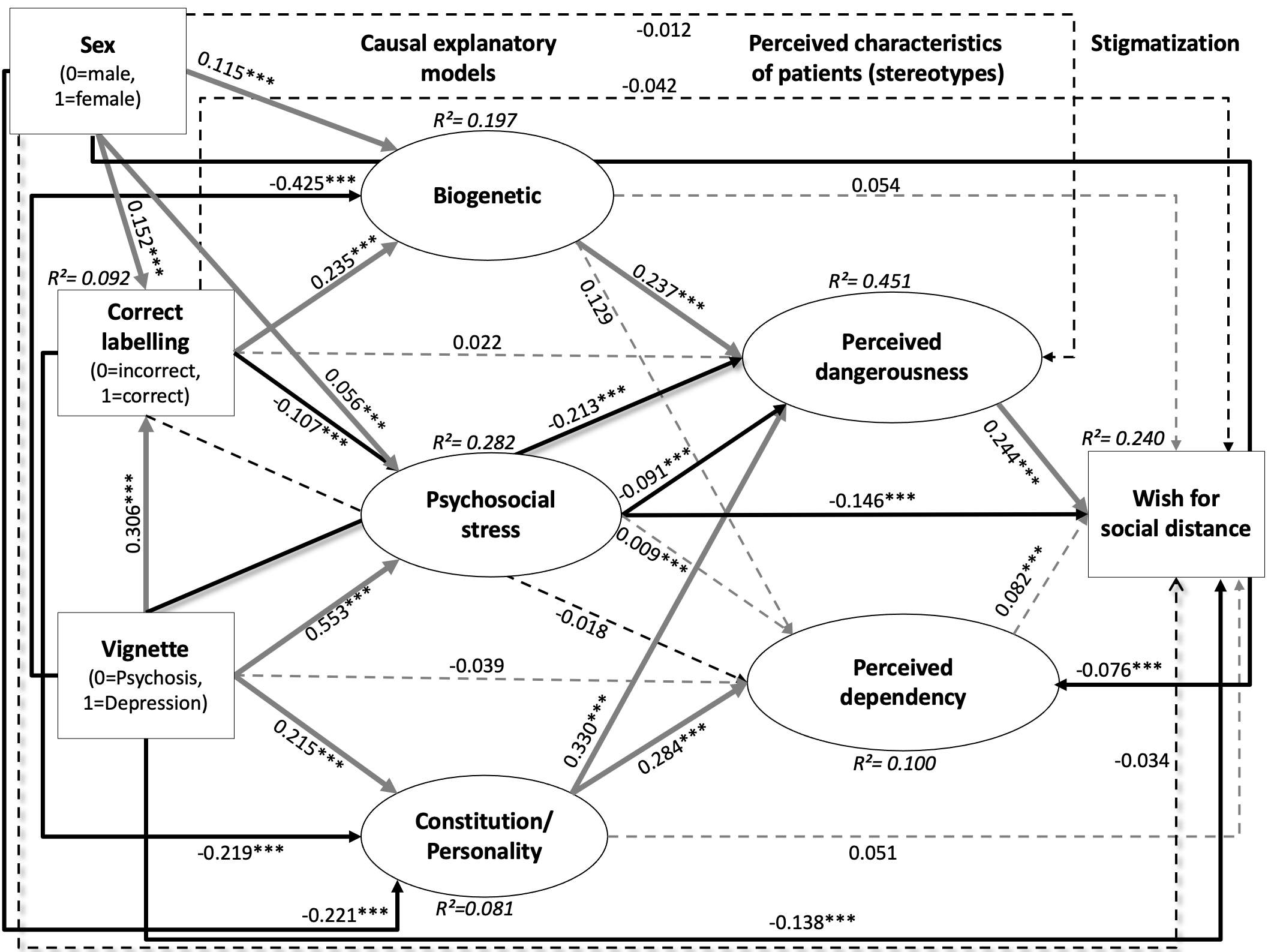


eFigure 3. Model of associations between five causal explanation variables, stereotypes and WSD and sex as a control variable (n=1526).

Model fit indices: χ^2^_(376)_= 2627.016 with p<0.001, CFI= 0.860; SRMR= 0.051; RMSEA= 0.057 (90%CI= 0.055 - 0.060); PNFI= 0.692.

Note: ***p≤0.001; standardized path coefficient; explained variance (R^2^) for each endogenous variable in italics. Manifest variables are represented in rectangles. Latent variables are represented in ovals. Solid lines indicate significant paths, dashed lines indicate; in doing so, grey indicates positive, black negative correlations.
