# Supplementary material for: The Important Role of Stereotypes in the relation between Mental Health Literacy and Stigmatization of Depression and Psychosis in the Community
Source: Community Ment Health J. 2021 May 26;58(3):474–86. doi: 10.1007/s10597-021-00842-5 (PMC8860791; doi:10.1007/s10597-021-00842-5)
Supplement: Supplementary file 5 — Supplementary file5 (DOC 49 kb) [file 10597_2021_842_MOESM5_ESM.doc]

eTable 1. Sample characteristics of responders and eligible non-responders/refusers to the add-on questionnaire study (N=2539).

|  | **Responders**  (n=1526) | **Refusers/Non-Responders**  (n=1013) | **Statistics**  U/χ²(df); Pearson’s r/ Cramer’s V |
| --- | --- | --- | --- |
| **Sex, n (%) male** | 718 (47.2) | 628 (61.6) | χ²_(2)_=66.945, p<0.001,  V= 0.158 |
| **Age: median (mean ± SD)** | 33.9 (31.3 ± 7.3) | 31.8 (29.8 ±7.8) | U = 693 553, p < 0.001,  r=-0.025 |
| **Nationality, n (%) Swiss** | 1455 (95.7) | 927(91.0) | χ²_(2)_=25.946, p<0.001, V=0.098 |
| **ISCED 2011^a^, n (%)** |  |  | χ²_(4)_=53.633, p<0.001,  V= 0.100 |
| Primary education (ISCED 1) | 17 (1.1) | 17 (1.7) |  |
| Secondary school (ISCED 2) | 828 (54.5) | 694 (68.1) |  |
| High School (ISCED 3) | 675 (44.4) | 308 (30.2) |  |
| **Employment, n (%)** |  |  | χ²_(8)_=52.878, p<0.001,  V= 0.099 |
| Unemployed, seeking employment | 25 (1.6) | 33 (3.2) |  |
| Protected employment | 3 (0.2) | 2 (0.2) |  |
| Temporary work, self-employed | 18 (1.2) | 7 (0.7) |  |
| Normal employment | 1474 (97.0) | 977 (95.9) |  |
| **Marital status, n (%)** |  |  | χ²_(10)_=20.316, p<0.001,  V= 0.062 |
| Unmarried | 799 (52.6) | 618 (60.6) |  |
| Married or registered partnership | 668 (43.9) | 359 (35.2) |  |
| Separated | 20 (1.3) | 15 (1.5) |  |
| Divorced | 29 (1.9) | 25 (2.5) |  |
| Widowed | 2 (0.1) | 1 (0.1) |  |
| Other | 2 (0.1) | 1 (0.1) |  |
| **Current non-psychotic axis-I disorder^b^, n (%)** | 192 (12.6) | 134 (13.2) |  |
| **Family member with a mental disorder, n (%)** |  |  |  |
| Affective disorder | 401 (26.4) | 189 (18.6) | χ²_(2)_=22.955, p<0.001,  V= 0.093 |
| Psychotic disorder | 48 (3.2) | 21 (2.1) | χ²_(2)_=2.737, p=0.254,  V= 0.032 |

^a^ according to International Standard Classification of Education (ISCED) (UNESCO Institute for Statistics, 2012).

^b^ according to Mini-International Neuropsychiatric Interview.
