# Supplementary material for: The Important Role of Stereotypes in the relation between Mental Health Literacy and Stigmatization of Depression and Psychosis in the Community
Source: Community Ment Health J. 2021 May 26;58(3):474–86. doi: 10.1007/s10597-021-00842-5 (PMC8860791; doi:10.1007/s10597-021-00842-5)
Supplement: Supplementary file 6 — Supplementary file6 (DOC 34 kb) [file 10597_2021_842_MOESM6_ESM.doc]

**eTable 2.** Examples for coding the item correct labelling.

| **Type of vignette** | **Correct labelling** | **Incorrect labelling** |
| --- | --- | --- |
| **Depression** | Affective disorder  Mood disorder  Depression  Depressive, depressed | Mental disorder  Psychiatric disorder  Bipolar disorder  Anxiety disorder  Personality disorder  Over-burdened  Burn-out  Having a personal problem  Having a mental problem  Sadness, sad  Mood swings  Midlife or life crisis  Stress  Low mood  Out of touch with reality  Madness, mad  Disoriented  Confused |
| **Schizophrenia** | Psychosis, psychotic  Psychotic disorder  Paranoia, paranoid  Schizophrenia  Hallucination  Delusion |
