# Supplementary material for: The Important Role of Stereotypes in the relation between Mental Health Literacy and Stigmatization of Depression and Psychosis in the Community
Source: Community Ment Health J. 2021 May 26;58(3):474–86. doi: 10.1007/s10597-021-00842-5 (PMC8860791; doi:10.1007/s10597-021-00842-5)
Supplement: Supplementary file 7 — Supplementary file7 (DOC 73 kb) [file 10597_2021_842_MOESM7_ESM.doc]

eTable 3. Standardized factor loadings and their corresponding standard errors of latent variables resulting from the SEM.

| Item of latent variable | Standardized factor loading | Standard error ^a^ |
| --- | --- | --- |
| **Causal explanations** |  |  |
| **Psychosocial stress** |  |  |
| Problems or sorrows in family | 0.677*** | - |
| An unconscious conflict | 0.375*** | 0.047 |
| Too high self-expectations | 0.618*** | 0.055 |
| Severe or very stressful life event | 0.516*** | 0.042 |
| Daily hustles | 0.785*** | 0.049 |
| Too high self-expectation | 0.618*** | 0.055 |
| **Biogenetic** |  |  |
| Brain disease | 0.847*** | - |
| Heredity | 0.450*** | 0.064 |
| **Constitution/Personality** |  |  |
| Weak constitution | 0.447*** | - |
| Weak will | 0.777*** | 0.155 |
| Immoral lifestyle | 0.515*** | 0.103 |
| God’s will | 0.256*** | 0.063 |
| **Substance abuse** |  |  |
| Alcohol abuse | 0.435*** | - |
| Medication or drug abuse | 1.422 *** | 1.119 |
| **Childhood adversity** |  |  |
| Little support others | 0.342*** | - |
| Grown up in a broken home | 0.688*** | 0.196 |
| Spoiling or over-protective parents | 0.454*** | 0.133 |
| Lack of parental affection | 0.791*** | 0.225 |
| **Personal attributions** |  |  |
| **Dangerous/Unpredictable** |  |  |
| Unpredictable | 0.531*** | - |
| Uncontrollable | 0.699*** | 0.042 |
| Aggressive | 0.594*** | 0.038 |
| Strange | 0.636*** | 0.047 |
| Scary | 0.713*** | 0.048 |
| Dangerous | 0.700*** | 0.039 |
| **Dependent/Needy** |  |  |
| Needy | 0.240*** | - |
| Helpless | 0.562*** | 0.640 |
| Dependent on others | 0.704*** | 0.570 |
| *** p<0.001  ^a^ fixed to 1.0 for the first selected item | | |
